# The Surgery of the Spinal Cord

**Published:** 1891-06-20

**Authors:** 


### SURGERY.

### THE SURGERY OF THE SPINAL CORD.

To those who watch the developments of surgery the importance of this branch of it must have been long apparent, nor are there wanting signs that this importance is duly recognised; and the scope for surgical aid in the treatment of spinal injuries and diseases unfolds itself more and more. That it is full of disappointments is only what is to be expected, not only because it is in its infancy, but because of the inherent peculiarities and difficulties which belong to its field of operation, and these should not dishearten us. The bearing of these difficulties, at least in one department of spinal surgery, is ably pointed out by Mr. Thorburn in his book on spinal injuries, and, although we do not share the extremely pessimistic view he is constrained to adopt, the material he has collected deserves our most careful study. With regard to the two tests of operative treatment—the risk incurred and the benefit to be expected—as applied to this branch of surgical work, we will quote what was said by Mr. Victor Horsley at the Berlin Congress last year: "I have now trephined the spine (opening the theca in six cases) nineteen times with one death, that is, from shock. The death-rate (if of any value or interest) is, therefore, a little over 5 per cent. Personally, I view the operation in the majority of cases as possessing the only risk of sepsis . . . and in view of the inevitable fate of such cases not so treated it is certainly *une quantité négligeable*. As to benefit, I venture to claim all improvement for the operation, inasmuch as retrogression and death otherwise infallibly occur." What this "improvement" amounts to in the various conditions that call for such interference we shall see.

I. *Spinal Meningocele.*—The usual and the best treatment probably is aspiration and injection, but Robson and others have excised the sac with a good result, and Horsley advocates this treatment if the simpler method fails.

II. *Neureotomy of the nerve roots for localised spasms or severe pain.*—Horsley treated two cases, in one with "much relief," and in the other with only "partial relief," other nerve roots becoming subsequently invaded. Mr. W. H.

Bennett recorded a case of acute spasmodic pain in the lower extremities, which was "completely relieved" by such a measure. Unfortunately, death occurred after apparent convalescence on the twelfth day, and *post-mortem* a clot was found over the left occipital lobe.

III. *Tumour of the Cord.*—In June, 1887, Mr. Horsley removed a myxoma of the cord, which produced complete paraplegia with great pain—the patient being reported as "perfectly well" in July, 1890. His second case—that of a growth surrounding the theca for four inches—died from shock.

IV. *Compression Paraplegia.*—This is one of the members of the group that is being brought most prominently before the medical world just now. In 1889 (*British Med. Jour.*, April 20th), Mr. Lane published a case of angular curvature —with paraplegia—in a boy of feeble constitution, in which he resected three laminæ with the result of a complete recovery, and last week he publishes two fresh cases—one of which "has recovered complete voluntary control over her legs." Mr. Lane has operated in all on eight cases, all the patients being in a feeble state, often with concomitant tuberculous disease elsewhere, of which five are apparently permanently relieved of their paraplegic symptoms, one has relapsed, one shows no improvement, and one died from hæmorrhage from a rectal polypus while the spinal condition was progressing satisfactorily. Such a table as this is most promising, there being no death referable to the operation, and only two cases in which relief was not obtained.

In 1890, at the Berlin Congress, Mr. Horsley tabulated seven cases similarly treated; one completely recovered, and another in which the motor power gradually returned could "walk" a year later; three were slightly improved (of which one died in six weeks from exhaustion), and only one is reported as being *in statu quo*. If, then, we add this table to the last, we get a series of fifteen cases with seven recoveries (nearly 50 per cent.), and no death directly due to the operation. Such results are an ample justification for surgical interference.

V. *Paraplegia from fracture of the spine.*—These are the cases which are such a heart-sore to the surgeon, and where repeated failures of any treatment to avert the "inevitable fate," which sooner or later awaits the unhappy sufferer, make the most hopeful of us at times despair. This is the feeling which seems to be uppermost in Mr. Thorburn's mind as the "conclusion of the whole matter," but from this we feel bound to dissent. The statistical table is here our curse. What is one failure after another in such a case if at last one success comes to crown our efforts? And we may be thankful that here and there a complete success is achieved to stimulate our flagging hopes. The case published by Mr. Golding-Bird two weeks ago will come as such a refreshing stimulus to many of us. Horsley published six cases, in one of which sensation and some movement were recovered, and which is reported in July, 1890, as "still improving." But all his cases (with the possible exception of one where no time is mentioned) were old-standing ones. In this connexion, therefore, it may be well to quote what Mr. Golding-Bird says on this point: "Inasmuch as nerve fibres, when divided, begin within three days to degenerate, laminectomy for spinal fracture should be undertaken within that period, for though paralysis from pressure of the cord may exist for much longer and yet be recovered from, thus showing the pressure to have been not destructive of the fibres of the cord; yet if we wait long enough to judge of the severity of the lesion by the disappearance or not of the paralytic symptoms, we shall certainly have waited too long for operation to offer a chance of success if the latter of these results unfortunately supervenes."

This we consider is a plea for early interference convincing enough, and, although many cases, unfortunately, are beyond the reach of any operation to relieve, more especially those (unhappily the majority) where the fracture is due to indirect violence and the cord is practically cut across by one part of the spine being displaced forwards over the other part, it teaches us that if we would save some we must not waste time. And here a symptom noted by Bastia will come to our aid. If *total* loss of sensation and motion and of reflexes below the seat of fracture persist for more than twenty-four hours (thus eliminating spinal shock), then the cord is completely disorganised at the seat of lesion, and no operation can avail anything. But, failing this significant sign, wherever the history of the accident is obscure, and the result of examination does not clearly indicate what is producing the pressure, we agree with Mr. Golding-Bird that an exploratory operation should be urged upon the patient in the hope that the condition may be one admitting of relief.

